# Nanofiber-Enabled Rapid and Non-Destructive Sensors for Meat Quality and Shelf-Life Monitoring: A Review

**DOI:** 10.3390/foods14223842

**Published:** 2025-11-10

**Authors:** Karna Ramachandraiah, Elizabeth M. Martin, Alya Limayem

**Affiliations:** 1 Department of Biological Sciences, College of Arts & Sciences, University of North Florida, Jacksonville, FL 32224, USA; karna.r@unf.edu; 2 Food Science, Department of Biological and Agricultural Engineering, University of Arkansas, Fayetteville, AR 72701, USA; emartin@uark.edu

**Keywords:** nanofibers, sensors, innovative films, freshness indicators, meat quality, real-time, electrospinning

## Abstract

The meat industry faces significant economic losses and environmental impacts due to spoilage and waste, much of which results from inadequate, delayed, or inefficient quality assessment. Traditional methods used for assessing meat quality are often time-consuming, labor-intensive, and lack the ability to provide real-time information, making them insufficient for modern supply chains that demand safety, freshness, and minimal waste. Recent advances in nanotechnology position nanofibers (NFs) as promising materials for addressing these challenges through smart sensing and active packaging. NFs, characterized by their high surface-to-volume ratio, tunable porosity, and small diameter, enable superior encapsulation and immobilization of sensing agents. These features improve the efficiency of colorimetric indicators, electronic noses, biosensors and time–temperature indicators. Electrospun NFs functionalized with metallic nanoparticles can detect contaminants such as antibiotics and hormones, while polymeric NFs embedded with reduced graphene oxide act as electrodes for advanced biosensing. Freshness indicators based on pH and nitrogenous compounds demonstrate real-time spoilage detection through visible color changes. This review explores nanofiber fabrication methods, their integration into sensing systems, and their potential to advance rapid, sustainable, and cost-effective meat quality monitoring.

## 1. Introduction

Meat quality refers to the characteristics and attributes that determine if meat is suitable for immediate consumption or can be stored for an extended period without deterioration [1,2,3].The attributes are mainly determined based on nutritional parameters (e.g., protein, lipids, minerals), meat hygiene/safety parameters (e.g., microbiological parameters, contaminants, adulterants), processing parameters (e.g., drip loss, shear force value, pH, connective tissues) and sensory parameters (e.g., taste, odor, texture, color, marbling) [4,5]. However, meat deteriorates due to three main mechanisms: microbial, lipid oxidation and enzymatic degradation. Therefore, preservation approaches focus on control of water activity and temperature and inclusion of bioactive agents/preservatives (e.g., nitrites, organic acids, chlorides, sulfides) [6,7]. Moreover, to preserve the quality and extend the shelf life of meat products, it is essential to employ appropriate handling, pretreatment, and preservation methods. However, during processing, distribution and storage, many factors can contribute to the shelf-life of meat. Meat products are influenced by extrinsic factors that include storage type, temperature control, packing system and quality management systems [6,8] Although the traditional methods (chemical and instrumental techniques) used for the quality determination of meat products are considerably more effective than manual inspection, these methods are laborious, time-consuming and cost-intensive. Therefore, there is a need for non-destructive, early and rapid detection methods to determine quality deteriorations in meat products [2,9].

Quality perception (by consumers) of meat products is contingent upon signaling of important information (related to product quality) at the point of purchase and while preparing and consuming the product. Furthermore, as consumers are seeking rapid and easy to prepare foods, food industries have created product categories: ready-to-prepare items, ready-to-heat, ready-to-cook, and ready-to-eat [10,11]. These categories are produced through the effective utilization of technologies and smart packaging. While high-pressure processing and pulsed electric fields have been exploited to produce safe products, packaging encompasses the use of labels or sensors, certificates and sustainable composite materials [8,10]. Since packaging material can not only influence food quality but also quality perception, new innovative packaging materials including nanomaterials are being developed [10,12]. Importantly, studies have focused on packaging integrated with sensors or indicators that assess the freshness of packaged food products including meat in real time [13,14]. As a result, sensors have been developed that possess rapidity, specificity, reliability, selectivity, cost-effectiveness, portability and non-destructive detection [15,16]. In addition to rapid assessment and monitoring of freshness, sensors are being investigated for the detection of contaminants and adulterants [17]. However, in food quality monitoring, the main categories of sensors typically employed include colorimetric sensors, electronic noses (e-nose/e-tongue), biosensors and time–temperature indicators (TTI) [18]. The colorimetric sensor market is projected to grow at a compound annual growth rate (CAGR) of 4.56% from 2025 to 2034, with a projected value of USD 2.85 billion [19]. The biosensor market has an estimated CAGR of 6.52% with a projected growth of USD 42 billion at the end of 2030 [20].

With growing interest in sensors, various new materials, including nanomaterials, are being actively investigated [13,14]. Recent technological advances have significantly enhanced the development of multifunctional nanofibers [8,21]. Nanofibers are filamentous structures with diameters smaller than 1 μm [22]. Nonetheless, 2D or 3D nanofibers can be fabricated that, in addition to having a high surface-area-to-volume ratio, exhibit high porosity and interconnectivity, high encapsulation efficiency, and strong thermal and chemical stability [8,23]. Moreover, nanofibers can be utilized to fabricate biodegradable films that serve as sustainable alternatives to conventional plastic packaging [24]. NFs facilitate efficient entrapment and encapsulation of diverse payloads (e.g., dyes, enzymes, bioactive agents) while allowing for controlled release based on NF characteristics such as porosity and diameter. NFs have also been incorporated as fillers into films/packaging formed by other polymers, thereby improving the barrier (gas, moisture), mechanical (tensile strength (strength), elongation at break (flexibility), Young’s modulus (stiffness) and even optical properties of the composite films/packaging [25,26]. Similarly, NFs can also form free standing films/mats with improved flexibility, transparency and higher encapsulation efficiency. Apart from efficient entrapment and encapsulation of various payloads, its release can be controlled by varying the NF characteristics such as porosity and diameter [27]. While numerous studies have explored sensors for food quality analysis, research on novel sensors based on nanofibers, particularly for meat quality assessment, remains limited. Therefore, this review describes NF production methods, meat deterioration mechanisms, factors and quality markers. This review also explores the rapid assessment of freshness, contaminants and adulterants in meat products using the major types of sensors.

## 2. Meat Loss and Waste

**Figure 1:**
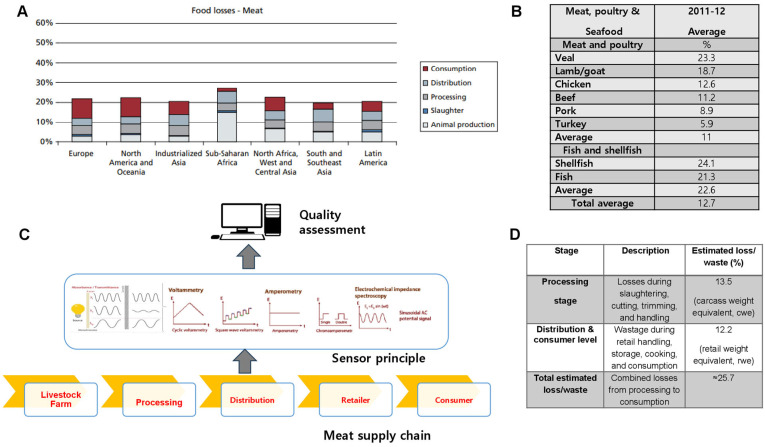
Global losses and wastes across meat supply chain. (**A**) Regional meat loss distribution. The bar chart illustrates total meat losses across major world regions at different stages of the supply chain: animal production, slaughter, processing, distribution, and consumption. (**B**) Average meat and seafood losses. Veal and lamb/goat exhibit the highest loss rates among meats, while shellfish shows the greatest waste among seafoods. The overall average loss across all products is ~12.7%**.** (**C**) Sensor-based quality monitoring. Diagram showing electrochemical and spectroscopic sensing (voltammetry, amperometry, impedance spectroscopy) for real-time freshness assessment across the meat supply chain. (**D**) Losses by processing and distribution stage. About 13.5% of losses occur during processing and 12.2% during retail and consumption, totaling ~25.7%, highlighting the need for improved preservation and monitoring technologies. Adapted from [17,28,29,30,31].

Food losses must be minimized regardless of a nation’s economic development, as they can directly or indirectly affect the income of both consumers and producers. In developing countries, losses are observed at the production stage of the supply chain, while in developed nations, losses are more commonly seen at the consumption end. More specifically, losses and waste in the US and Europe are proportionally higher at the retail and consumer levels (Figure 1A). The comparatively lower waste in developed nations is due to lower animal mortality at the production and transportation stages, whereas higher waste is attributed to higher animal mortality (due to diseases) in developing countries [28].

Using the most recent OECD-FAO Agricultural Outlook model, it was estimated that, globally, approximately 13.5% of total carcass meat is lost during slaughtering and processing, while a further 12.2% of retail-ready meat is wasted during distribution and consumption [29]. Additionally, reducing meat waste can help lower environmental impacts by decreasing the carbon footprint as well as water and energy use [28]. Figure 1B shows the greater losses observed with seafood compared to meat and poultry [30]. As noted in an earlier study by Buzby et al. [31], higher losses are due to various factors, such as the increase in the types of meat products available at supermarkets. Furthermore, food products have been classified as ready-to-heat, ready-to-cook, ready-to-prepare and ready-to-eat [10]. Owing to the diverse products, stores must manage both shelf space and the inventory of the meat products [30]. To this end, sensors can play a crucial role for retailers, as they help monitor and manage the inventory of perishable products more efficiently, ensuring optimal shelf space utilization and reducing waste. While meat waste and loss also impact the environment, this is not discussed in this review.

## 3. Meat Quality Deterioration

**Figure 2:**
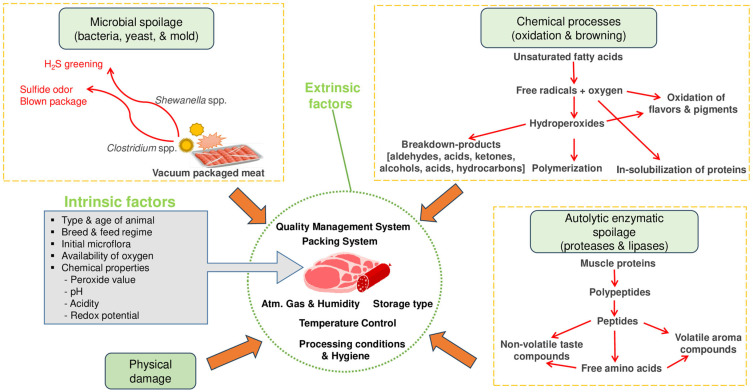
Meat spoilage mechanisms and factors affecting meat quality. Adapted from [6].

Meat products primarily deteriorate due to microorganisms (bacteria, yeast, and mold), enzymes (proteases and lipases), and chemical processes (oxidation and browning) (Figure 2). While physical damage can also occur, meat spoilage is commonly caused by microorganisms [32]. However, the auto-oxidation of fatty acids and generation of free radicals have also been identified as contributors to meat product spoilage. Lipid oxidation results in hydroperoxides, which are further degraded into secondary reaction (lipid oxidation) products such as aldehydes (malondialdehyde, 4-hydroxynonenal, hexanal, ketones and acids) [33]. Fat deterioration can also occur through certain enzymes (lipases, esterase and phospholipase), which can be endogenous or originate from specific (psychotropic) microorganisms. On other hand, the breakdown of protein has been attributed to autolytic enzymes (calpains, cathepsins and aminopeptidases) [6]. Meat products contain a wide range of microorganisms that include pathogens. Earlier studies have shown that the most common bacteria are *Enterococcus* spp., which have been found on various types of meat and refrigerated products. Other bacterial species reported are *Clostridium, Pseudomonas*, *Lactobacillus*, *Streptococcus*, *Escherichia*, *Salmonella*, *Sarcina*, *Micrococcus*, and *Bacillus*. Major mold species found on meat products are Penicillium, Cladosporium, Mucor Sporotrichum, and Geotrichum. Examples of yeasts species include *Rhodotorula* spp., *Cryptococcus* spp., and *Candida* spp. [3,34].

The preprocessing (preproduction) factors that influence the quality or shelf life of meat products are related to animal type (bovine, ovine), age, genetics, sex, pre-and post-slaughter conditions (chilling, hygiene, quality management), initial microflora, chemical properties (water activity, pH, peroxide value, redox potential, acidity), and availability of oxygen [6,35]. Among the intrinsic factors, age has been reported to have a greater impact on tenderness than breed. In the case of external factors, chilling is known to strongly influence the microbiological, technological and sensory attributes of meat [35,36]. During processing or production of meat products, several factors that include margination, smoking and cooking methods play an important role. Ingredients (e.g., salt, spices, herbs) and additives (preservatives, colorants, flavor enhancers, binders, emulsifiers) also influence the major quality attributes of meat products [37,38]. In the storage and distribution stage, external parameters that affect the quality of meat products include quality management systems, temperature, humidity, aerobic systems (anaerobic/vacuum, aerobic, MAP), and packing systems (materials, equipment, gases) [6].

## 4. Meat Quality Markers

Some of the major markers used to indicate the quality of meat products include color, temperature, humidity, gases (O_2_, CO_2_), pH changes and nitrogenous compounds [39]. Temperature in an essential indicator/marker which influences the shelf life of meat products. In pork products, a higher population of Enterobacteriaceae was observed when placed at higher temperatures (15 to 25 °C), whereas Acinetobacter was more prominent than Enterobacteriaceae when products were placed at temperatures >30 °C [40]. As a result, time–temperature sensors/indicators have been developed that quantify and monitor the impact of temperature on food products [18]. Oxygen is known to cause oxidation of lipids, whereas carbon dioxide is reported to prevent the growth of microbes (bacteria, fungi), which is due to lowered pH and the creation of an anaerobic environment. Therefore, assessment of the concentration and type of gas provides information on the quality of meat products. To this end, O_2_ and CO_2_ detectors have been employed for their detection in food products [39]. The production of organic acids (acetic, formic, lactic acid) has been linked to lowered pH, which is detected using various types of sensors. However, in refrigerator conditions, the growth of lactic acid-forming bacteria (LAB) is retarded and they are outnumbered by other bacteria (Pseudomonads). Hence, in aerobically packaged meat, LAB is rarely found, whereas this bacterium is commonly found in vacuum or modified atmosphere packaged meat. Similarly, humidity is also reported to affect the shelf life of meat products with an increase in Pseudomonad numbers under humid conditions. Humidity sensors have been integrated with radio frequency identification (RFID)-based tags for real-time assessment of humidity [39].

The major markers released by microorganisms consist of various compounds such as sulfur and nitrogenous compounds, esters, ketones, alcohols, and aldehydes. These compounds have shown a strong correlation with microbial growth and lower sensory acceptance of different types of meat [13]. The protein degradation products and volatile nitrogen compounds (mainly ammonia, dimethylamine and trimethylamine) are evaluated using the total volatile basic nitrogen (TVB-N). Likewise, biogenic amines (putrescine, cadaverine, histamine, tyramine), which are formed by decarboxylation of amino acids, also indicate the spoilage of meat products. Common methods used for TVB-N quantification include Kjeldahl and FTIR analysis. Methods used to identify food spoilage markers through chemical analysis involve techniques such as liquid chromatography, tandem mass spectrometry, and various spectroscopic approaches. Volatile compound analysis methods include gas chromatography and mass spectrometry [41].

## 5. Nanofiber Preparation Methods

Nanofibers for food applications have been derived from natural (protein, polysaccharides) and synthetic (PVA, PEO, PLA) polymers. However, nanofibers used in the development of sensors have been derived from diverse materials including cellulose (Plants, bacteria), chitosan, gelatin, carbon, metals, ceramics, and metal oxides [42]. Nanofibers produced by physical methods include ultrafine grinding, micro-fluidization, homogenization, phase separation, cryo-crushing and ultra-sonication [27,43]. Chemical methods (e.g., enzymatic, acid hydrolysis, TEMPO-facilitated oxidation), which are also often used in conjunction with some physical methods, are also commonly used in the production of NFs [44,45]. However, in recent times, electrospinning has attracted immense attention due to its simplicity, cost-effectiveness, capacity to regulate fiber size/diameter and scalability of production. Recent studies have indicated that electrospinning (solution or melt) offers a more cost-effective route for nanofiber fabrication than traditional melt spinning, which depends on mechanical extrusion of molten polymers through spinnerets [46]. As a result, electrospinning is widely adopted in the various areas including the development of innovative packaging and sensors [27,47]. Various NF fabrication methods are shown in Table 1.

Electrospinning primarily involves applying an electric field to a polymer solution to produce nanoscale fibers [48]. In the first stage, high voltage is used to generate electrostatic force. The flow of spinning fluid/polymeric solution is controlled by a flow control pump. When an electrical force is applied, the charged polymer solution in the spinneret needle (syringe with needle) deforms into a Taylor cone, from which the jet elongates and the solvent evaporates, resulting in the formation of solid nanofibers [48,49]. Fibers are drawn out and collected on the collector when the electrical force surpasses the fluid’s surface tension at a critical voltage. Nanofibers can be produced using different electrospinning techniques, including needleless electrospinning, nanospider electrospinning, coaxial electrospinning, and multi-jet electrospinning [49]. NFs can also be produced using other methods (non-electrospinning) such as self-assembly, Interfacial Polymerization, and Spinneret-Based Tunable Engineered Parameters (STEPs) [49]. A variety of synthetic polymers (e.g., polycaprolactone, polyvinyl alcohol, and polyacrylonitrile), as well as natural polymers like zein, gelatin, and chitosan, along with their combinations, can be used in the electrospinning process [50,51]. The electrospinning parameters (applied voltage, flow rate, and collecting distance) can be optimized for each polymer system based on solution viscosity and environmental conditions (temperature and humidity). Several studies have discussed various experimental protocols and parameter values for electrosppining NFs [27,52]. Nonetheless, the electrospun NFs can incorporate various types of payloads including drugs, enzymes, bioactive agents, microorganisms, dyes and pigments [53].

**Table 1:** Different NF fabrication methods, underlying mechanisms, merits and demerits.

Fabrication Method	Underlying Mechanism	Key Benefits	Limitations	References
Template Synthesis	Fibers form within a template, which is later removed.	Allows fabrication of fibers with varying diameters using different templates.	Challenging template removal process.	[8]
Drawing	Mechanical stretching of polymer melts or solutions into fiber.	Requires basic equipment.	Challenging to achieve fibers smaller than 100 nm. Intermittent production.	[8,27]
Self-assembly	Molecular interactions drive the spontaneous organization of NFs. (e.g., Peptide NFs)	Simple method for creating multifunctional nanofibers.	Complex processing. Limited polymers. Increased production cost. Minimal production efficiency.	[8,27]
Electrospinning	Uses a high-voltage electric field to draw polymer solution into fine fibers.	Produces nanometer- to micrometer-sized fibers. Cost-effective. High aspect ratio. Enhanced mechanical properties.	Inconsistent fiber formation. Restricted pore size tunability. Dependence on hazardous solvents.	[27,50]
Phase Separation	Polymer phase separation creates porous NF structures.	Suitable for batch production. Enables controlled pore size and structure. Requires minimal equipment.	Restricted to specific polymers. Unsuitable for lengthy fibers.	[27,50]
Centrifugal Spinning	Uses centrifugal force to stretch polymer solutions into fibers.	Increased throughput rate. Straightforward. Cost-effective.	Challenges in fiber collection.	[50,53]

## 6. Effect of Structure, Dimensions, and Morphological Properties

Compared to other nanostructures, such as spherical nanoparticles (NPs), nanofibers (NFs) exhibit different payload (drug) release mechanisms. According to a study, nanofibers (NFs) generally facilitate a controlled and sustained release through diffusion, whereas nanoparticles (NPs) tend to exhibit an initial burst release, which can negatively impact effectiveness and reproducibility [54,55]. To minimize burst release, researchers have explored strategies such as surface modifications and cross-linking of matrices [54]. Additionally, core–shell electrospun fibers have been developed to enhance sustained release, where the payload is enclosed within a polymeric core and coated with an outer layer [56]. Compared to NPs, NFs demonstrate a more controlled release profile, often following close-to-zero-order kinetics [57].

The morphological properties of NFs are influenced by various factors during electrospinning, including polymer concentration, solvent (viscosity, surface tension) characteristics, conductivity, and molecular weight. Adjusting processing conditions such as flow rate, applied voltage, humidity, and air velocity can refine NF diameter, porosity, and mechanical strength [27,58]. For example, increasing polymer concentration improves fiber formation, whereas volatile solvents enhance porosity [59]. In particular, porous NFs loaded with dyes have shown enhanced reactive molecules and active sites. However, loading indicator dyes into the polymer solution decreases its viscosity, which, in turn, results in the formation of beads. Therefore, it is important to consider these factors when designing NFs [47]. Similarly, high-pressure homogenization plays a role in determining NF crystallinity, yield, and size by adjusting pressure and cycle count [60]. Nonetheless, due to their distinctive properties, such as nanoscale diameters and an exceptionally high surface area, electrospun nanofibers provide significant advantages over traditional films produced through solvent casting or extrusion techniques [47].

## 7. Rapid and Real-Time Monitoring and Evaluation of Quality

The quality of meat is traditionally linked to the sensory perception (appearance, tenderness, flavor, color, and odor) of whole meat as opposed to meat products. However, in the following sections, quality of meat products is expressed as freshness and wholesomeness. Fresh/wholesome meat products are products that are devoid of pathogens, infectious agents and toxic products [61].

### 7.1. Freshness Assessment via Colorimetric Indicators

Meat quality analysis traditionally relies on methods such as HPLC, MS, TLC, and capillary electrophoresis, which are time-consuming and costly. To overcome these challenges, sensors are being developed for real-time, in situ meat quality assessment [15,62]. While computer vision and multispectral analysis provide precise, non-destructive evaluation, they require complex optics and extensive data processing [63]. As an alternative, freshness indicators are integrated into packaging as on-packaging sensors or indicator films [64]. Recently, these sensors have gained attention for their simplicity (visible color changes), adaptability to various polymers, and cost-efficiency [65]. The most commonly used freshness indicators function based on pH fluctuations. These indicators rely on the reaction of sensing agents (dyes or pigments) with specific metabolites or target molecules (NH_4_, CO_2_, H_2_S, organic acids, volatile amines, biogenic amines), resulting in observable color changes. The sensing (detection) agents include pH responsive pigments (such as curcumin, anthocyanin, and shikonin) and dyes (including alizarin, carminic acid and tartrazine). Preferably, natural sensing agents with low or no toxicity are incorporated into natural polymer-based matrices (e.g., cellulose, chitosan, chitin, starch, gums) to form biodegradable, sustainable films [8]. The effectiveness of freshness indicators depends on multiple factors, including the type and concentration of sensing agents, the polymer matrix, and the presence of additives [66]. However, these indicators face challenges such as limited discoloration range, colorant instability, and potential leakage. To overcome these limitations, nanofibers (NFs) are integrated into composite films and labels, enhancing the absorption of sensing agents onto the film’s surface and improving overall effectiveness [66]. This enhanced functionality is attributed to the high surface area and abundant hydroxyl groups in NFs, which serve as a polymeric foundation for the incorporation of indicator (detection) compounds [67].

### 7.2. On-Packaging Indicators

Indicators are directly integrated into packaging materials or used as labels, stickers, inserts, films, or coatings [68]. Indicator labels/films based on pH have been fabricated for quick detection of meat freshness in real time. The color changes in the on-packaging label are readily discernible by the naked eye [62]. While indicators (on-packaging) may require direct contact with food for improved performance [62], leaching or migration of components could result in health risks. Hence, using safe indicator materials or placing the labels/films in the headspace of the packaging has been recommended [69,70]. In the study by Li et al. [69], an indicator film composed of electrospun poly-L-lactic acid (PLLA)/anthocyanin changed from pink to colorless when exposed to ammonia. The limit of detection for ammonia was 35.39 ppm. In another study, on-packaging labels were fabricated with bacterial cellulose (BC) NFs and anthocyanin (pigment) for the detection of freshness in packaged fish carp fillets (Table 2) [71]. BC comprises a 3D uni-axially oriented network of fibers produced by bacteria such as *Komagataeibacter xylinus.* Bacterial cellulose has been reported to possess greater crystallinity (> 60%) than plant-based cellulose [72]. Anthocyanins are pigments derived from plants that change color in reaction to metabolites. However, in this study, anthocyanin was coated onto commercial BC NFs (64 ± 12 nm) using an ex situ method, dip coating. The label (anthocyanin loaded BC NFs) showed a diverse spectrum of colors depending on the pH (2–11). In a packaging containing fish (carp) fillets, the labels fixed onto the headspace changed colors as the fillets deteriorated. The transition from carmine to pink to khaki indicated the stages of freshness, optimal consumption, and spoilage of the fish fillets. The sensing process was accelerated by the interconnected network (porous), which accelerated the diffusion of spoilage gases [71].

In a 2023 study, Jang et al. developed pH-sensitive indicator films using cellulose nanofibers (NFs) and red-radish color extract (RRCE) to assess minced pork quality. The cellulose NF-RRCE films, made through solvent casting, were placed in the headspace of containers with minced meat. The films changed color from light red (fresh) to red-purple (spoilt) as the pH increased due to the formation of volatile nitrogenous compounds from meat degradation. The cellulose NF matrix effectively dispersed the anthocyanin colorants, preventing agglomeration, due to hydrogen bonds between the NFs and colorant molecules [73]. While labels have been fabricated via in situ and ex situ methods to immobilize and entrap sensing agents onto nanofibers, electrospinning enables the encapsulation of one or more of the sensing agents within various polymers [65]. In an investigation by Duan et al. [74], a stable film was fabricated using pullulan and chitin nanofibers (NFs) embedded with anthocyanin and curcumin. The addition of two sensing agents (anthocyanin and curcumin) increased the diameter from 177 nm to 380 nm; however, the structure of the NF remained unaffected. Films (1 × 1 cm) formed using the electrospun NFs were attached to the lid of the Petri dish containing fish (*Plectorhynchus cinctus*) samples. Owing to its pH sensitivity, the NF-based film was used to monitor the spoilage of fish. The combination of sensing agents maintained the color changing effect with color changes from pink to blue (powdery) indicating spoilage [74]. However, a major demerit of films made with biopolymers such as chitosan and pullulan is their disintegration upon getting wet. Hence, to counter this drawback, crosslinking by either heat or by adding glutaraldehyde or a more environmentally friendly alternative, cinnamaldehyde, has been recommended [75].

In another study, Sun et al. [65] developed biogenic amine-sensitive labels using anthocyanin-loaded electrospun poly-l-lactic acid (PLLA) NFs. The addition of anthocyanin did not affect the NFs’ structure, which maintained a small diameter and uniform thickness. Electrospinning allows the use of solvents like DMF and DCM, which influence the solution’s polarity, controlling NF diameter and uniformity. Smaller NF diameters enhance surface area, porosity, and sensing performance. The labels, when placed in the headspace of a Petri dish with mutton, changed from pink to colorless, indicating freshness and spoilage. These biodegradable polymer labels showed high stability across temperatures (4 to 25 °C). However, as noted by van der Schueren et al. [76], leaching of dyes from NFs could occur which could be countered by adding complexing agents (e.g., poly (diallyldimethylammonium chloride)). To improve sensing performance, films have been doped with nanoparticles such as TiO_2_ and Silver nanoparticles (AgNPs). Han et al. (2023) [77] synthesized labels using PVA-based nanofibers (NFs) combined with AgNPs and grape seed anthocyanidin to monitor pork freshness. The inclusion of grape seed anthocyanidin reduced the average NF diameter due to increased crosslinking but enhanced porosity. The label’s color changed from light gray to dark gray, reflecting pork freshness, with results matching TVB-N values from chemical analysis. Similarly, Yildiz et al. [78] fabricated chitosan-PEO nanofiber films loaded with curcumin that changed color when exposed to the headspace of chicken breast packaging stored at 4 °C.

Visual detection of meat freshness has been achieved using fluorescent sensors, though they often suffer from low sensitivity. Recent progress has resulted in the creation of highly sensitive NF-based ratiometric fluorescent sensors capable of detecting biogenic amines (BA) at 1 ppm. Cellulose nanofibers (NFs) synthesized via TEMPO oxidation formed the sensor’s backbone, with fluorescein isothiocyanate (FITC) as the indicator and protoporphyrin IX (PpIX) as the internal reference [16]. Earlier fluorescent sensors faced issues with the detachment and release of fluorescent reagents. To address this, fluorescent compounds have been grafted onto matrices [79]. However, greater grafting (degree) of fluorescent compounds onto cellulose NFs was associated with increased sensitivity (low detection limit). Furthermore, the nanofiber skeleton improved air permeability, which is a major factor influencing the sensitivity of sensors. As the shrimp samples deteriorated, the fluorescent sensors (labels) affixed to the Petri plates consisting of shrimps were able to detect biogenic amines (BAs). The fluctuations in the fluorescence intensity (FITC present on NFs) in response to BA caused changes in color. As the concentration of BA increased, the color of the sensor changed from red to yellow green, a trend also observed with pork samples. The results were consistent with other meat quality parameters such as ΔE, TVB-N and total microbial count. Hence, cellulose NFs are regarded as ideal for creating simple fluorescent sensors that offer high sensitivity and selectivity [16].

Another indirect way of ensuring meat freshness is via the use of sensors that detect oxygen. In a study, meatball packages with a controlled atmosphere (0.1% oxygen) were used. Films were made using electrospun polyvinyl alcohol (PVOH) NFs containing glycerol, titanium dioxide (TiO_2_), and methylene blue (MB). Furthermore, films coated with polystyrene (PS) were placed inside packaging before sealing with 100% nitrogen gas. The indicator films were then UV-bleached and stored at 4 °C for 10 days. Throughout storage, the films remained colorless, confirming the package integrity. Upon opening, they turned blue due to oxygen exposure, with non-coated indicators showing weaker color recovery, likely due to increased humidity. The first equation (shown below) represents the reduction of MB (oxidized) by TiO_2_ and an electron (generated by TiO_2_ when exposed to UV light), resulting in a colorless form. The second equation shows the oxidation of MB (reduced) to MB+ in the presence of oxygen, resulting in a blue color.

(UV)                                 

↓                                   

2TiO_2_ (e^−^) + H^+^ + MB (Oxidized) → 2TiO_2_ + MB (reduced)…………. (Colorless)

2MB (Reduced) + O_2_ → 2MB^+^ (Oxidized) + 2OH^−^ ……………………. (Blue)  

This study shows the reliability of oxygen indicators in meat packaging, reinforcing their usefulness in detecting packaging damage and ensuring food quality [80].

### 7.3. Indicator Packaging

While polymer-derived indicator films/labels, which are attached to meat packaging, are utilized for the visual detection of meat quality in real time, innovative packaging has been developed that also serves as a quality indicator. Various types of NFs have been fabricated that are used either as matrices or as fillers/composite materials within matrices derived from other polymers [73,77]. BC NFs were incorporated into a film/matrix derived from konjac glucomannan (KG), a polysaccharide. The KG films produced by the casting method were supplemented with curcumin (a pH-sensitive sensing agent) alongside BC-NFs. To evaluate the freshness, beef samples were packaged with the KG-based films. The films loaded with 3% curcumin showed the best results, with red color indicating complete deterioration of the beef samples. Furthermore, the thermal stability of the film was ascribed to the hydrogen bonds that formed between the NF-KG matrix and curcumin [81]. Likewise, innovative indicator bags were created by combining cellulose NFs (as fillers), anthocyanin (as the sensing agent), and cellulose acetate (as the matrix) using the casting method. At a concentration (optimal) of 1.5%, cellulose-based NFs exhibited stronger binding between NF and anthocyanin. This was due to the electrostatic interactions between the functional groups of the NFs and the color-producing molecules. The indicator bags packaged with meat and fish samples changed colors from pink to dark bluish green as the meat samples deteriorated. The insertion of cellulose NFs in the matrix caused increased absorption and uniform distribution of anthocyanin (at optimal concentration), thereby increasing the functionality of the films [66].

Sensing agents have been embedded in films developed by casting methods or in mats fabricated by the electrospinning method. However, sensitivity of the packaging material is reported to be dependent on the fabrication method. In a recent study by Pereira at al. [82], gelatin-derived films as well as mats were fabricated by the casting method and electrospinning, respectively. The incorporation of anthocyanin influenced the structure of both the films and mats owing to the increased interaction between anthocyanin and polymeric matrices. The feasibility of mats and films for the quality evaluation of minced beef was assessed. The gelatin mats (alone) and gelatin–anthocyanin mats had higher sensitivity values of 54% and 45% compared to gelatin films (alone) and gelatin-–anthocyanin films, respectively. The highest sensitivity was observed with gelatin–anthocyanin mats, with a detection level of 150–300 ppm of ammonia at room temperature (25 °C). Furthermore, the results showed a strong correlation with the pH and TVB-N values. As the electrical response was quantified using a potentiostat, it was suggested that the electrospun mats are more suitable for freshness assessment of meat products. Hence, electroactive sensors based on NFs could be fabricated with the natural polymers and compounds, precluding the need for toxic metal or metal-oxide NPs [82].

A major problem with indicator films made with pH-sensitive compounds is the gradual leaching out of the colorant from the packaging into food [83]. To counter this issue, bilayer films have been developed that act as a preventive barrier while providing stability to the indicator films. Zhang et al. [84] synthesized an NF-based bilayer film for visual monitoring of pork quality. The electrospun bilayer film consisted of an antibacterial layer (polyvinylidene fluoride and vanillin) and sensor layer (polyvinyl alcohol, alizarin, sodium alginate). While vanillin provided the antibacterial effect in the antibacterial layer, alizarin acted as the sensing agent in the sensor layer. As a result, the shelf life of the pork samples (stored at 25 °C) was increased by 24 h, whereas the color changed from yellow at 0 h (fresh) to dark purple (spoilt) after 96 h. Therefore, electrospun NFs can be utilized to fabricate bilayer packaging films (antimicrobial and indicator) that provide improved shelf-life and real-time monitoring of meat products [84]. In recent years, growing attention has been directed toward the development of edible and biodegradable electrospun films, such as zein–theaflavin (Z/TF) composites, which actively enhance food preservation. These natural polymer-based materials not only reduce plastic waste but also incorporate bioactive compounds that improve antioxidant and antimicrobial protection, thereby extending product freshness and safety [51]. Similarly, hybrid electrospun nanofiber films prepared from gelatin and green tea extract powder (HGGTNF) have shown strong antioxidant and antimicrobial activities, significantly reducing lipid oxidation, microbial counts, and drip loss in beef compared with polyethylene films [24]. However, the incorporation of sensing materials into such edible packaging has yet to be explored.

**Table 2:** Nanofiber-based freshness indicator labels (on-packaging) and built-in indicator packaging.

Nanofiber Type	Sensor Type	Sensing Agent	Meat Product Type	Visual Changes	References
Cellulose NFs	Indicator label	Fluorescein isothiocyanate	Shrimps	Red (fresh) to yellow green (spoilt)	[16]
Poly-l-lactic acid (PLLA)	Indicator label	Anthocyanin	Mutton	Pink (fresh) Colorless (spoilt)	[65]
Cellulose NFs	Freshness indicator bags	Anthocyanin	Meat and Fish	Pink (fresh) Bluish green (spoilt)	[66]
Bacterial cellulose NFs	Indicator label	Anthocyanin	Rainbow trout and common carp fillets.	Carmine (fresh) Pink (best to eat) Khaki (spoilt)	[71]
Cellulose NFs	Indicator label	Red-radish color extract	Minced pork	Light red (fresh) purple (spoilt)	[73]
Pullulan and chitin nanofibers (NFs)	Indicator film	Anthocyanin Curcumin	Fish	Pink to blue (powdery)	[74]
Polyvinyl alcohol (PVA) NFs	Indicator label	Grape seed anthocyanidin	Pork	Light pink (fresh) Grey (spoilt)	[77]
Polyvinyl alcohol NFs coated with polystyrene	Oxygen indicator	Titanium dioxide (TiO_2_) and methylene blue (MB)	meatball	Colorless (intact packaging) Blue (damaged package)	[80]
BC NFs	Freshness indicator Packaging	Curcumin	Fresh Beef	Yellow (fresh) Red (spoilt)	[81]
Polymeric NFs	Freshness indicator and active packaging (bilayer)	Alizarin	Pork	Yellow (fresh) Dark purple (spoilt)	[84]

### 7.4. Freshness Assessment via Electronic Nose (E-Nose)

In addition to colorimetric sensors, the electronic nose (e-nose), an olfactory visualization sensor, has also been used to detect meat spoilage. E-nose simulates the mammalian olfactory system in the detection of volatile compounds (e.g., nitrogenous compounds, alcohols, ketones, esters) formed by the spoilage of meat [63]. Recently, e-tongue and e-eye, which mimic human senses of taste and vision, have been investigated in food and pharmaceutical industries for the quality control and shelf-life evaluation of various products [85]. However, e-noses have been widely used for rapid, simple and non-destructive detection of food quality through the quantification of compounds present in the sample headspace. The data derived from an e-nose cannot be directly used in the quantification of aroma-causing compounds. With the use of suitable mathematical techniques (neural networks or multivariate statistics), valuable aroma patterns are derived, which are in turn used to quantify analytes [86]. Nonetheless, the e-nose is based on the interactions, such as adsorption and van der Waals, between analytes and sensors [87].

An e-nose typically consists of gas sensors based on metal oxide semiconductors. While metal oxide semiconductors provide cost-effectiveness, a facile synthetic process, short response time, and chemical stability, they lack selectivity (concurrent reactivity to both oxidizing and reducing gases) [88]. To address this limitation, researchers have focused on functionalizing the surfaces of metal oxide semiconductors or developing hybrid systems. André et al. [14] investigated semiconducting metal oxide–based nanomaterials (NPs, nanowires, and NFs) for gas detection using electrospun SiO_2_:In_2_O_3_, SiO_2_:ZnO, and SiO_2_ nanofibrous mats.The three different types of NFs were embedded onto electrodes (interdigitated) using polyaniline. Similarly, another set of sensing units weas prepared by replacing polyaniline with polystyrene sulfonate. The sensing (gas) performance of the different units was determined by measuring the electrical impedance. The impedimetric e-nose was able to distinguish and monitor volatile amines (ammonia, methylamine and trimethylamine) in fish meat. The higher surface-area-to-volume ratio of the metal oxide nanostructures is known to improve the performance of the e-noses. However, fish meat placed in a plastic container was attached with the sensor mat (array) [14]. E-noses involve an array, which combines different resistive sensors. This is because a sensor giving out a single value is considered non-selective. In particular, the output value of resistive sensors is calculated by taking the ratio of a target gas (R_g_) to its resistance in air (R_a_). The selectivity of sensors relies on its capacity to differentiate two types of gases (oxidizing and reducing), which cause either an increase or decrease in the resistance. Consequently, multiple resistive sensors are employed in the development of an array for the recognizing different patterns [89]. Nonetheless, in the study by Andre et al. [14], sensor array demonstrated superior performance, and a multidimensional projection method was used to calculate the complex AC impedance data that was derived from the sensor array. In addition, the 3D fibrous interconnected inorganic mats were self-supportive, precluding the need for a substrate. Therefore, it was suggested that the e-nose (free standing strips) could be employed for the rapid and on-site detection of meat freshness. However, some studies have reported that the e-nose has higher sensitivity for temperature and humidity compared to colorimetric sensors [63].

### 7.5. Freshness Assessment via Biosensors

Biosensors are a category of sensors that consist of a physicochemical transducing system integrated with biomaterials such as whole cells, tissues, nucleic acids, organelles, aptamers and enzymes [90]. The major types of commonly used transducers are optical, electrochemical, piezoelectric (mass based) and thermistors (thermal-based). When the target molecule (analyte) in the test sample interacts with the bio-component (biomaterial), it generates an electric signal that can be easily quantified and recorded. The optical systems have widely used silicon wafers covalently bonded to protein [91]. The electrochemical biosensors also have three major types based on the measurement of electrical parameters such as amperometric, conductometric and potentiometric. While amperometric sensing involves the quantification of a current that has resulted from a redox reaction, potentiometric sensing quantifies the potential difference (voltage) between two electrodes. Conductometric sensing is related to the measurement of electrical resistance or conductance of a sample [92]. However, in food analysis, biosensors have been used in the detection of various analytes including small proteins, organic molecules and inorganic compounds [91]. Biosensors have attracted increased attention due to their potential for on-site application, rapid detection, lowered costs and the ability to be easily miniaturized [93].

Biosensors have been used to detect leakage in packaging (moisture, O_2_, CO_2_) and indicators of food product spoilage such as gaseous amines, volatile organic compounds (ethanol, acetone, CO), ethylene gas (indicator of fruit ripening), hypoxanthine, xanthine and uric acid [92]. In many types of seafood (fishes and shellfish), the amount of adenosine triphosphate (ATP) and its breakdown products like xanthine, hypoxanthine, and uric acid serve as indicators of freshness. During the initial phases of storage, endogenous enzymes cause the degradation of ATP into hypoxanthine (Hx) through many intermediates such as adenosine diphosphate (ADP), adenosine monophosphate (AMP), inosine monophosphate (IMP), and inosine (INO). Further degradation of seafood causes the conversion of HX into xanthine (Xa) and uric acid. While IMP is considered a flavor enhancer (e.g., meaty, umami), HX is reported to contribute to off flavors [94]. Moreover, the accretion of metabolites (HX, Xa) in stored (chilled) seafood is used as an indicator of freshness. Thus, to overcome the demerits (cost- and time-intensiveness) of conventional methods (HPLC and spectrophotometry) of metabolite (HX and Xa) evaluation, novel biosensors have been investigated [95].

To detect the freshness of chilled seafood (squid and large yellow croaker), an electrochemical biosensor was constructed using a biological component: xanthine oxidase (XOD) (Table 3) [95]. The enzyme was immobilized on a NF film composed of copper-based metal organic framework nanofibers (CuMOF). Metal organic frameworks (MOFs) are nanoscaled three-dimensional coordination networks composed of organic linkers and inorganic connectors. In the study by [95], MOF NFs were precipitated by mixing organic ligands with metal salt solutions in an aqueous medium. MOFs (non-conducting) were incorporated onto conductive NFs containing copper (Cu). The CuMOF, which was immobilized with XOD, was dispersed in sodium alginate solution. This mixture was drop-cast onto a surface to form films. The XOD-based biosensor exhibited high sensitivity toward hypoxanthine and xanthine, achieving low detection limits of 0.0023 μM and 0.0064 μM, respectively. The stable NF film was able to effectively entrap XOD allowing for optimal transfer of electrons between the enzyme and electrode. Therefore, it was suggested that the amperometric biosensor could be used for the rapid and highly sensitive detection of seafood freshness [95].

In another study, amperometric biosensors were fabricated with cobalt (benzene 1, 3, 5-tricarboxylic acid) MOF deposited onto porous carbon (electro-spun) NFs via the solvothermal deposition method. The free standing (sea urchin-like) biosensors were prepared for the detection of Xa and uric acid in salmon fish fillet. The Co(TMA)MOF-Carbon NF electrode showed superior selectivity and sensitivity in the detection of Xa and uric acid. The limits of detection for Xa and uric acid were 96.2 nM and 103.5 nM, respectively. Polyacrylonitrile (PAN) was electrospun to form NFs, which were carbonized at a high temperature of 800 °C (under nitrogen flow), resulting in a carbon NF mat. The porous NFs (diameter of 280–300 nm) and Co(TMA)MOF hybrids were able to form 3D sea urchin-like structures, which facilitated the effective attachment of analytes and allowed the transfer of electrons and ions, thereby improving its electro-catalytic performance. In addition to improved electron transport kinetics, carbon NF-based mats (electrosun) are reported to be superior to other nanomaterials due to their capacity to form highly flexible electrodes [96]. Other merits associated with electrospun NFs are their ease of recyclability and higher diffusion of substrate owing to increased porosity, which make them more suitable for immobilization of enzymes [97].

### 7.6. Pathogen Detection by Sensors

Electrospun nanofiber-based biosensors have been fabricated for pathogen detection in various types of food. Pathogens such as *L. monocytogenes*, *P. putida*, *S. aureus*, *E. coli*, and *Salmonella* have been identified in different types of meat [98]. *Salmonella*, a rod-shaped Gram-negative bacterium (Enterobacteriaceae family), is estimated to cause about 1.4 million cases of salmonellosis each year in the United States, primarily through food transmission [99]. In one study, *Salmonella* was detected in milk using an aptasensor developed with a pencil graphite electrode (GE) embedded with chitosan-electrospun carbon nanofibers (CNFs) and gold nanoparticles (NPs). The chitosan-carbon NF coating (90 nm) on the electrode surface improved analyte binding, enhancing sensor performance. Key optimization parameters included pH, sonication time, incubation time, and CNF volume. The optimal volume of 3 µL gave the best results, while increasing the volume compressed the NF, reducing surface area and current. The sensor’s limit of detection (LOD) was 1.223 CFU/mL, significantly lower than PCR’s 102 CFU/mL [100].

*Salmonella* has been detected in fresh beef samples using a sensor linked to a microfluidic chip, resulting in a lab-on-chip device. In a study by Thiha et al. [101], suspended carbon nanofibers formed electrodes for a chemiresistive biosensor platform. The carbon nanowires (84 nm), functionalized with an aptamer (*Salmonella* specific), enabled quick detection of *S. Typhimurium* within 5 min. The total assay time, including incubation and measurement, was significantly shorter than PCR (3 h) and the graphene aptasensor (10 min) [102,103]. The bacterial suspensions for detection were derived by homogenizing and culturing bacteria in Rapport Vassiliadis broth (42 °C for 24 h). However, this method theoretically does not require pathogen culturing or DNA extraction, unlike PCR and LAMP methods. The assay demonstrated high sensitivity and specificity, requiring only 5 µL of sample, with a limit of detection (LOD) of 10 CFU/mL [101].

Although not tested on meat, nanofiber-based sensors have been used to detect *E. coli* O157:H7 and *Listeria monocytogenes*. The FSIS considers *E. coli* O157:H7 an adulterant in ground beef, prohibiting its sale [104]. *L. monocytogenes* contamination is often due to poor sanitation during meat processing, particularly when transitioning from cooking to packaging [105]. In a study, *E. coli* O157:H7 as well as bovine viral diarrhea virus (BVDV) were detected using a conductometric lateral flow biosensor. The sensor’s capture pad, made of electrospun nitrocellulose nanofibers, provided a high surface area for antibody binding, improving pathogen capture. Plasma treatment of the nanofibers enhanced their hydrophilicity. The nanofibers also ensured effective capillary action for rapid sample flow and uniform distribution, promoting the formation of a sandwich complex between the pathogen and immobilized antibodies, which generated an electrical signal. The biosensor detected pathogens in 8 min with limits of 61 CFU/mL for bacteria and 103 CCID/mL for viruses. Electrospinning and surface functionalization enabled efficient, cost-effective nanofibrous capture membranes for pathogen detection [106]. In an earlier study, a microelectrode-based immunosensor for detecting *Listeria monocytogenes* was developed using TiO_2_ nanowires synthesized via a hydrothermal reaction. The nanowires were attached to gold microelectrodes, with monoclonal antibodies restrained on their surface to selectively capture the bacteria. Impedance changes from the nanowire−antibody−bacteria complex were measured, correlating with bacterial concentration. This sensor detected *L. monocytogenes* as low as 102 CFU/mL in 50 min, with minimal interference from other pathogens [107].

As the detection of pathogens (low concentrations) in meat products is important for safety prediction, colorimetric sensors have been fabricated that detect bacterial contamination. Electrospun NFs (poly-l-lactic acid (PLLA) and anthocyanin) ensured rapid, uniform color changes, allowing for the detection of low bacterial concentrations (102 CFU/mL) in a non-destructive headspace environment [69]. Although the study focused on bacterial detection using LB agar plates, this technology has strong potential for application in meat quality analysis. The film’s ability to sense bacterial growth through volatile metabolic compounds could be adapted for real-time monitoring of spoilage in packaged meat and seafood.

**Table 3:** Biosensors composed of NFs for freshness and pathogen detection.

Nanofiber Type	Sensor Type	Sensing Agent	Meat Product Type	Results/Applications	References
TiO_2_ nanowires	Impedance Immunosensor	Monoclonal antibodies	Meat samples	Detection of Listeria monocytogenes in meat 102 CFU/mL	[71]
Copper-based MOF nanofibers (CuMOF)	Electrochemical Biosensor	Xanthine Oxidase (XOD)	Squid and large yellow croaker	Detection of hypoxanthine and xanthine in seafood freshness assessment LOD 0.0023 and 0.0064 μM,	[95]
Cobalt MOF-carbon nanofiber	Amperometric Biosensor	Cobalt (benzene 1, 3, 5-tricarboxylic acid) MOF	Salmon fish fillet	Detection of xanthine and uric acid 96.2 nM (Xa), 103.5 nM (Uric Acid)	[96]
Chitosan-carbon nanofibers with AuNPs	Voltammetric Aptasensor	Aptamer	Milk	Detection of Salmonella (1.223 CFU/mL)	[100]
Carbon nanofibers	Chemiresistive Biosensor	Aptamer	Fresh beef	Rapid detection of Salmonella Typhimurium 10 CFU/mL	[101]
Nitrocellulose nanofibers	Conductometric Lateral Flow Biosensor	Antibodies for E. coli O157:H7 and BVDV	Food samples	Detection of E. coli O157:H7 and BVDV 61 CFU/mL (bacteria), 103 CCID/mL (viruses)	[106]

### 7.7. Time–Temperature Indicators (TTI) Based Quality Assessment

TTIs are sensors that track, record, and display the cumulative effect of temperature on a food product’s quality across the supply chain [47]. These simple, inexpensive sensors, when integrated into food packaging, provide information about a specific temperature and time. As a result, TTIs have been widely utilized to monitor the shelf-life of seafood [108], meat [109], and fish products [110] at specific temperatures [111]. A range of TTIs, including microbial, enzyme-based, polymeric, solid-state reaction, and diffusion-based systems, are widely used. Among these, enzyme TTIs are particularly effective due to their high sensitivity to temperature changes, surpassing microbial and diffusion-based alternatives [112]. Common enzymes like amylase, laccase, lipase, and urease have been utilized in TTI designs, though challenges such as cost and instability limit their widespread use. As a result, diffusion-based TTIs dominate the market [113]. To improve enzyme stability and enhance sensitivity, researchers have explored immobilizing enzymes in solid-state matrices, such as electrospun fiber mats, which also help reduce enzyme consumption. Additionally, these mats offer potential for improving other TTI systems [47].

Tsai et al. [114] fabricated an electrospun TTI mat for monitoring milk spoilage by immobilizing laccase on mats (1 cm^2^) made of chitosan/polyvinyl alcohol/tetraethylorthosilicate NFs. The TTI changed color from transparent to purplish brown over storage at 4 °C, with deeper coloration at higher temperatures and enzyme dosages. An enzyme dosage of 8–10 g/cm^2^ effectively indicated lactic acid bacteria (LAB) growth, responding to LAB reaching 10^6^ CFU/mL in milk. In an earlier study, a microbial TTI made using nutrient broth, Chlorophenol red solution, and *Lactobacillus sakei* strain was used to monitor spoilage in modified atmosphere packed (MAP) minced beef. The detection involved pH changes caused by the growth and metabolic activity of the *L. sakei* strain, with a corresponding color change in the indicator from red to yellow [115]. Polymeric TTIs composed mainly of paraffin wax and black carrot anthocyanin have also been used to detect temperature-induced spoilage in fish products by showing color changes in response to pH variations [116]. While several studies have utilized TTIs to monitor meat spoilage, none have incorporated nanofibers. NFs can be utilized to enhance the performance of TTIs due to their high surface area and ability to support the incorporation of various sensing agents (such as enzymes, dyes, or other materials). The use of nanofibers can improve the sensitivity, stability, and responsiveness of the TTIs to temperature changes, making them more efficient in monitoring food quality or spoilage over time.

## 8. Sensor-Based Detection of Contaminants and Adulterants

Apart from evaluating meat deterioration, nanomaterials have been used to detect contaminants (antibiotics, drugs, hormones, pesticides) and adulterants (e.g., nitrites or meat from other animals) in meat. Antibiotics are commonly used in livestock farming for disease treatment. While antibiotics can promote weight gain in animals, their overuse has led to antibiotic resistance, which is a growing concern [117]. Specifically, doxycycline hydrochloride (which belongs to the tetracycline class of antibiotics) and enrofloxacin (classified as a fluoroquinolone antibiotic) are frequently used in poultry to control pathogens. These antibiotics were detected in chicken meat samples using polyvinyl alcohol (PVA) nanofibers (NFs) decorated with gold nanoparticles (AuNPs) through Surface-enhanced Raman Spectroscopy (SERS) [118]. SERS is a vibrational spectroscopic technique known for its rapid, high-sensitivity, and non-destructive analysis. Among various flexible platforms for SERS substrates, electrospun nanofibers have shown significant promise [119]. The functionalization of these substrates with metal nanoparticles creates hotspots that enhance detection sensitivity by at least 102 times compared to conventional Raman scattering [120]. In the study by Sarma et al. [118], electrospun PVA NFs mixed with AuNPs were used as SERS substrates, improving the identification of antibiotics in chicken meat samples. The antibiotic quantities determined by the SERS method were validated using liquid chromatography–mass spectrometry (Table 4) [118]. The decoration of NFs with metallic nanomaterials can also be accomplished via an ex situ method. In a study by Chen et al. [121], AgNPs were embedded onto NFs electrospun with polyvinyl alcohol (PVA) and polyethyleneimine (PEI). After electrospinning the PVA-PEI NFs, AgNPs were adsorbed onto the fibers electrostatically. The Ag-adsorbed PVA-PEI NFs served as a Surface-enhanced Raman Spectroscopy (SERS) substrate for detecting enrofloxacin in prawn samples [121]. Enrofloxacin is a fluoroquinolone antibiotic used to control pathogens in fish. Since some fish drugs, like enrofloxacin, furazolidone, and malachite green, can be toxic and contribute to bacterial resistance, their use has been banned in some countries. However, their use persists in many developing countries [122].

Fishes have also been reported to contain microcystins, a type of cyanotoxin that represents one of the primary routes of microcystin exposure to humans [123]. In a study by Xu et al. [124], electrospun nanofibers (NFs) significantly improved the detection of microcystins. The nanofibrous structure provides a high surface area and abundant active sites, enabling efficient immobilization of metal organic frameworks (MOFs), AuNPs, and aptamers for selective recognition. This architecture not only enhances binding affinity and extraction efficiency but also ensures rapid mass transfer and stability during analysis. As demonstrated, the nanofiber-based platform achieved ultrasensitive and highly specific detection of microcystins, far surpassing traditional extraction methods. In recent years, aptamers have gained increasing attention due to their high affinity and specificity [125]. Aptamers, which are single-stranded DNA or RNA molecules, have been used as alternatives to antibodies in sensor design. Recent innovations have led to the development of aptasensors integrated with various nanomaterials for detecting different target analytes. For example, aptasensors have been combined with MXenes [126] and quantum dots [127]. It has been shown that when aptamers were grafted onto MOF-functionalized electrospun nanofibers, toxin detection was considerably enhanced, achieving a limit of detection (LOD) of 0.004 ng/mL [124]. It is important to note that while some sensors require simple sample pretreatment, pretreatment-free sensing systems are more desirable.

Endocrine-disrupting chemicals (EDCs), including polycyclic hydrocarbons, hexachlorobenzene, polychlorinated dibenzodioxins, and polychlorinated naphthalenes (PCNs), have been found in eggs and meat products. These chemicals enter livestock primarily through pesticide use in farming [128]. EDCs can negatively impact human health, causing infertility and reproductive issues [129]. A previous study used bioassays to detect dioxin-like EDCs in the skin and extracts of beef, chicken, and fish [130]. Free-standing nanofiber (NF)-based sensors have been developed to detect endocrine-disrupting chemicals (EDCs). Polyacrylonitrile (PAN)-derived nanofibrous membranes were used to filter a reduced graphene oxide (rGO) dispersion, forming a freestanding PAN-rGO membrane. This membrane was created via electrospinning, and rGO synthesis involved hydrothermal processing of graphene oxide. The PAN-rGO membrane served as an electrode to quantify 17α-ethinylestradiol (EE2) by measuring electrical impedance. The limit of detection for EE2 ranged from 10 μmol L^−1^ to 10 pmol L^−1^. The NF-based sensor was able to distinguish between estrone, estradiol, and progesterone in river water samples [129]. However, the application of these sensors for detecting EDCs in meat samples has not yet been explored. This sensor type offers a straightforward, cost-effective approach with high sensitivity and selectivity for EDC detection.

Nitrites are commonly used as an additive in meat products such as ham, sausages and cured meat [120]. While this additive provides an excellent antimicrobial effect, particularly against *Clostridium botulinum,* it has been linked with increased risks of certain some types of cancers (colorectal cancer, stomach cancer) [37]. As a higher concentration of nitrites has been associated with the formation of carcinogenic nitrosoamines in meat products, the levels of nitrites are analyzed [131]. However, the chemical or instrumental analysis of nitrites is known to be time-consuming and expensive. Therefore, sensors are being studied for the rapid analysis of nitrites in various meat products. In a recent study, [120], NFs formed by electrospinning of a mixture of zein, acetic acid, gluten and glycerol. The NFs were then coated (electro-sprayed) with AgNPs to form SERS substrate. The limit of detection of this SERS substrate was estimated to be 15.29 ng·L^−1^. The Ag-coated NF-based SERS platform was able to detect nitrites in canned pork, ham, chicken sausages and cured meat. The nitrite contents of the fine extracts (devoid of fat and protein) of these meat products were comparable to the results obtained using the chemical method. Therefore, it has been suggested that the response layer based on Ag-coated NFs offers an exceptional platform for the accurate, cost-effective and rapid estimation of nitrites in various types of meat products [120].

Due to the relatively higher cost of certain meats (beef, mutton), some meat products have been adulterated with cheaper meat derived from animals (e.g., pork) which are considered unacceptable to certain individuals or groups. Therefore, to detect such adulterants in meat products [132], biosensors have been developed that are highly sensitive and cost-effective. In this regard, carbon NFs were functionalized by electro-grafting a 4-carboxyphenyl layer on their surfaces. The electro-grafted carbon NF-modified screen printed carbon electrodes were attached covalently with antibodies via amide bond. The biosensors formed using an in situ electrochemical method were able to detect porcine serum albumin (PSA), which is also considered as an allergen present in pork meats. The binding of PSA to antibodies caused variations in the cathodic current signal originating from the electrode. Due to the higher surface area of carbon NFs, the immobilization of antibodies and electronic conductivity were increased, thereby improving the sensing capacity. Moreover, the carbon NF electrode, used as a transducer, was combined with square wave voltammetry (SWV), an electrochemical technique to identify adulterants like pork in meat. With a low detection limit of 0.5 pg/mL, these carbon NF-based biosensors were suggested to be suitable for rapid on-location identification of adulterants in meat products [93].

**Table 4:** Nanofiber-based sensors for detection of contaminants and adulterants in meat products.

Nanofiber Type	Sensor Type	Sensing Agent	Meat Product Type	Results/Applications	References
PVA Nanofibers	Surface-enhanced Raman Spectroscopy (SERS)	Gold Nanoparticles (AuNPs)	Chicken	Detection of antibiotics (doxycycline, enrofloxacin)	[118]
Zein-based Nanofibers	Surface-enhanced Raman Spectroscopy (SERS)	Silver Nanoparticles (AgNPs)	Pork, ham, sausages	Detection of nitrites	[120]
PVA-PEI Nanofibers	Surface-enhanced Raman Spectroscopy (SERS)	Silver Nanoparticles (AgNPs)	Prawn samples	Detection of enrofloxacin	[121]
PAN-rGO Nanofiber	Electrical Impedance Measurement	Reduced Graphene Oxide (rGO)	Possibly in meat samples	Potential detection of endocrine-disrupting chemicals	[129])
Carbon Nanofibers	Electrochemical Sensor (SWV)	Antibodies (for porcine serum albumin)	Beef, mutton products	Detection of pork adulteration	[132]

## 9. Challenges and Future Perspectives

Developing on-site or on-package sensors that allow rapid detection, need little or no sample preparation, deliver high sensitivity and specificity, are cost-effective, and easy to use is essential. Multiplexing sensors that detect multiple analytes (chemical markers) instead of quantifying pathogens are preferable, as not all pathogenic isolates (e.g., Salmonella) are highly virulent [133,134]. Despite several studies showing superior performance, sensors are not widely used in food industries. The major impediments for the widespread use of sensors are production cost and scalability [135]. Some nanomaterials (e.g., carbon nanotubes, gold nanoparticles), which are essential for improved performance, are known to increase production costs [136]. To overcome these drawbacks, cost-effective nanomaterials that can withstand adverse conditions (e.g., low temperatures and high moisture) are needed. Additionally, to ensure the stability and integrity of sensors, further studies on improved coatings and packaging are required [137]. While certain sensor manufacturing methods (e.g., chemical vapor deposition, lithography) are considered less favorable [138], novel needleless electrospinning has been reported to have the potential for mass production [139]. To lower the production cost of nanofibers, prototype electrospinning devices have been developed that are considerably cheaper (USD 202) than conventional electrospinning devices (USD 15,000–60,000) [140]. Approaches to reduce costs include the utilization of homemade voltage sources, various types of rotating drum collectors, spinneret tips, and customized syringe pumps. Apart from the widely used single-needle setup, needleless and centrifugal-based electrospinning equipment has also been investigated for improved scalability [141]. Furthermore, disposable standalone sensors fabricated with sustainable materials need to be developed that can be integrated with cutting-edge assay techniques, smartphones and IoT [82].

## 10. Conclusions

Nanofibers, with their increased surface-to-volume ratio, smaller diameters, and adjustable pores, have found a wide range of applications in comparison to other nanostructures. Among the various fabrication methods, electrospinning is regarded as the most efficient, cost-effective and straightforward. Films or wraps or mats have been used to encapsulate or embed different types of active agents including sensing agents. Nanofiber-based indicator labels have been embedded or immobilized with sensing agent/compounds via in situ or ex situ methods. For rapid and real-time assessment, sensors have been fabricated to target quality markers (biogenic amines, gases, biogenic amines), temperature, humidity, pH changes and nitrogenous compounds in meat products. Freshness indicators commonly employed are colorimetric sensors, which are dependent on pH changes in meat products. Indicator labels attached to packaging could visualize spoilage in meat products, whereas indicator packaging serves both as an efficient packaging solution and as a rapid way of indicating freshness. Another visualization sensor, semiconducting metal oxide NFs (e-nose), has demonstrated improved capacity to detect volatile compounds in spoilt meats. In addition to freshness, contaminants such as antibiotics and hormones and adulterants such as inferior meats and nitrites, pathogens have been detected via NF-based innovative sensors. While polymeric NFs functionalized with metallic NPs have been used as SERS substrates for the detection of antibiotics, reduced graphene oxide embedded polymeric NFs have been employed as electrodes for the detection of hormones in meat products. Biosensors were prepared by immobilizing antibodies onto carbon NFs, which functioned as transducers in the detection of counterfeit (pork) meat. The NF-based membranes provide a higher surface area, allowing for enhanced adsorption, encapsulation and embedding of diverse nanomaterials. These membranes also function as freestanding sensors, resulting in improved sensor performance. Compared to other nanostructures, NFs offer promising applications in a wide range of sensors, enhancing the speed, sustainability and performance of meat quality analysis.

## Data Availability

No new data were created or analyzed in this study. Data sharing is not applicable to this article.
